# Another Woman Honored

**Published:** 1894-12

**Authors:** 


### Another Woman Honored.

Dr. Jessie M. Weston, one of the graduates of the Woman's Medical College of Philadelphia in 1893, has been elected to the medical staff of the Connecticut State Hospital for the Insane at Middletown, occupying the only medical position which the Nutmeg State government has thrown open to women.

